# The supply of general practitioners across local areas: accounting for spatial heterogeneity

**DOI:** 10.1186/s12913-015-1102-y

**Published:** 2015-10-03

**Authors:** Michelle McIsaac, Anthony Scott, Guyonne Kalb

**Affiliations:** Melbourne Institute of Applied Economic and Social Research, The University of Melbourne, 111 Barry Street, Carlton, VIC 3053 Australia

**Keywords:** General practice, Health care supply, Location choice, Spatial econometrics, Spatial heterogeneity, Redistribution policy

## Abstract

**Background:**

The geographic distribution of general practitioners (GPs) remains persistently unequal in many countries despite notable increases in overall supply. This paper explores how the factors associated with the supply of general practitioners (GPs) are aligned with the arbitrary geographic boundaries imposed by the use of spatially referenced GP supply data.

**Methods:**

Data on GP supply in postcodes within Australia are matched to data on the population characteristics and levels of amenities in postcodes. Tobit regression models are used that examine the associations between GP supply and postcode characteristics, whilst accounting for spatial heterogeneity.

**Results:**

The results demonstrate that GPs do not consider space in a one-dimensional sense. Location choice is related to both neighbourhood-specific factors, such as hospitals, and broader area factors, such as area income and proximity to private schools. Although the proportion of females and elderly were related to GPs supply, mortality rate was not.

**Conclusions:**

This paper represents the first attempt to map the factors influencing GP supply to the appropriate geographic level at which GPs may be considering that factor. We suggest that both neighbourhood and broader regional characteristics can influence GPs’ locational choices. This finding is highly relevant to the design and evaluation of relocation incentive programmes.

## Background

Access to primary care is a pervasive policy concern for most countries; however, increasing the supply of doctors does not necessarily lead to better access [1]. Some policies therefore also aim to address distributional issues rather than simply focusing on the overall supply of health care practitioners. With large regional variations in GP supply the United Kingdom (UK) and many European countries, such as Germany, have introduced policies and regulation aimed at discouraging GPs from locating in well-serviced areas [2]. Other countries, including Australia, have introduced financial incentive schemes and tailored training programs to encourage doctors to work in underserved areas [3].

GPs’ location decisions are influenced by a range of factors. If GPs care about profit as well as population health then the decision of where to practice is influenced by the relative levels of profit and health care need across areas, with the latter reflected by the demographics of the population. Area amenities (such as schools) and family characteristics (such as school-aged children) also matter if GPs are simultaneously choosing a place to live. Hence GPs’ preferences, family circumstances, as well as population and area characteristics can influence location choice.

Much of the literature assessing the factors associated with the distribution of the health workforce is dated. Using the number of GPs as the measure of GP supply, the literature concludes that the main variable determining the number of GPs in an area is population size [4–6]. Accordingly GPs are considered to be responsive to market forces; locating in areas where demand (i.e. population size) is highest. Other early literature suggests that income maximization, desire to live in high-status residential areas (or aversion to low-socioeconomic status areas), affluence of the service population, and proximity to private hospitals are factors that influence a GP’s location choice [7–9].

Studies assessing GP distribution attempt to account for health care need by including proxies such as the demographic characteristics of the population and various measures of health status. In these studies GP supply is measured as either a per capita ratio, or a needs-adjusted per capita ratio [10, 11]. These studies demonstrate that the number of places in nearby medical schools and average income (or house prices) are positively associated with GP supply; and that population demographic factors, such as age and gender, are also associated with GP supply [2, 12].

A major shortcoming of this earlier literature is that it largely ignores that the data are geographically referenced; that is, information is collected by geographical area, which are often arbitrary units of analysis that are not independent from proximate geographic areas. The geographical referencing inherent in GP supply data leads to several methodological issues that might influence the validity of results. These include the potential for omitting a variable that is spatially correlated, treating variables as independent when they are in fact related to the values of their neighbours, or using inappropriately aggregated data. The first two problems lead to econometric biases, whereas the third can, as pointed out by Birch et al. [13], lead to incorrect inferences regarding individual behaviour as associations may vary according to the level of aggregation of the data. Ignoring the geographical referencing of GP supply data can therefore lead to erroneous conclusions regarding the effectiveness of policies aimed at redistributing GPs.

This study aims to increase the understanding of the key drivers of GP distribution, by accounting for the geographical referencing of GP supply data. We explore how the factors associated with GP supply in Australia are aligned with the arbitrary geographic boundaries of GP supply data. The independent variables are analysed to determine their relationship to the same variables for their proximate neighbours. This paper represents the first attempt to map the factors influencing GP supply to the appropriate geographic level at which GPs may be considering that factor.

The Australian primary care sector is organised in a very similar manner to other primary care systems, particularly those with policies or funding which are linked to geographical boundaries. An important point of distinction is that it is funded using fee-for-service system with GPs able to charge patients what the market will bear. Unlike the UK, the Netherlands, Italy, Spain or Finland where patients are generally required to register with a GP, patients in Australia can visit any GP as there is no registration or enrolment. GPs’ location decisions are largely unregulated with almost no market entry restrictions. The exception is overseas trained doctors (OTDs) who have restrictions in place for the first 5-10 years after entry into the Australian market. These restrictions generally limit OTDs to working in a District of Workforce Shortage, which is generally a rural and remote area [14]. There are also education and training initiatives, such as increasing the amount of time spent in rural areas during training [15]. However, the most notable redistribution initiatives in Australia are the financial incentives aiming to encourage GPs to locate in rural and remote areas [16]. Over 130 million dollars (AUD) is currently (2009-2013) being targeted towards the rural health workforce strategy [15].

## Methods

### Statistical analyses

This paper examines how the non-pecuniary location-specific factors associated with GP supply are distributed across geographical space by estimating reduced-form associations of location attributes. Australian GP supply data at the level of the postal area is regressed on a set of area characteristics. The geographic nature of the data is taken into account by calculating the spatial correlation of all variables. This information is used to estimate two area-level regressions assessing the geographic level on which these variables are most strongly related to GP supply.

Due to the small geographic unit of analysis, several neighbourhoods exist without a GP; approximately 30 % of postal areas in Australia have no GP. This high frequency of zeros and the absence of negative GP supply result in data that do not have the properties of a standard normal distribution. Rather than an ordinary least squares regression, the model used accounts for the large number of zero outcomes in order to produce consistent and asymptotically normal estimates [17]. Maximum likelihood estimation is employed using the Tobit specification which allows for censoring at zero:

1 $$ {S}_i={\alpha}_0+{x}_i\beta +{u}_i,\kern0.5em \mathrm{where}\kern0.5em {u}_i\Big|{x}_i\sim Normal\left(0,{\sigma}^2\right) $$

where: *i* = postal area

*S*_i_^*^ = a latent variable underlying supply of GPs in the *i*^*th*^ postal area

$$ \left\{\begin{array}{c}\hfill {S}_i={S}_i^{*}\kern0.24em \mathrm{if}\ {S}_i^{*}\ge 0\ \hfill \\ {}\hfill {S}_i=0\kern0.36em \mathrm{if}\kern0.37em {S}_i^{*} < 0\hfill \end{array}\right. $$

*S*_*i*_ = supply of GPs in the *i*^*th*^ postal area

*x*_*i*_ = value of vector of independent variables in postal area of interest *i*

*α*_*0*_*, β* and *σ*^*2*^ = parameters to be estimated

Given that postal areas are an arbitrary delineation of space, a problem akin to that described in the geography literature as the modifiable areal unit problem occurs [18]. To address this, we permit GP supply in a given postal area to be related to factors not only in their postal area, but also neighbouring postal areas. Accounting for the spill-over effects between neighbourhoods reduces measurement bias in the model and accounts for the fact that GPs’ location choices could be influenced by variables at a more aggregated level than the neighbourhood where they are located. Spill-over effects between neighbouring areas have largely been ignored in the GP supply literature. Existing models implicitly assume that the proximity of the observations is not important. Taking into consideration spatial dependence, a spatially weighted model accounting for spatial heterogeneity is constructed by including variables to capture the characteristics of nearby neighbourhoods (i.e. spatially lagged variables).

Spatial heterogeneity is a concern when using spatially referenced data, therefore it is expected that some of the independent variables in this model will be spatially correlated.

The Moran’s I statistic (a measure of spatial correlation) is used to calculate the degree of spatial correlation in the independent variables. Moran’s I is expressed as:

2 $$ I=\frac{n}{{\displaystyle {\sum}_i{\displaystyle {\sum}_{j\ne i}{w}_{ij}}}}\frac{{\displaystyle {\sum}_i{\displaystyle {\sum}_{j\ne i}{w}_{ij}}}\left({x}_i-\overline{x}\right)\left({x}_j-\overline{x}\right)}{{\displaystyle {\sum}_i{\left({x}_i-\overline{x}\right)}^2}} $$

$$ \mathrm{where}:{w}_{ij}\kern0.5em =\kern0.5em \left\{\begin{array}{c}\hfill 1\kern0.5em \mathrm{if}\kern0.5em j\kern0.5em \mathrm{is}\kern0.5em \mathrm{a}\kern0.5em \mathrm{neighbour}\kern0.5em \mathrm{t}\mathrm{o}\kern0.5em i\hfill \\ {}\hfill o\kern0.5em  if\kern0.5em j\kern0.5em \mathrm{is}\ \mathrm{not}\kern0.5em \mathrm{a}\kern0.5em \mathrm{neighbour}\kern0.5em \mathrm{t}\mathrm{o}\kern0.5em i\hfill \end{array}\right\}\kern0.5em i=1, \dots,\ n;\ j = 1, \dots, n $$

$\overline{x}$ = mean of independent variables of all postal areas

*n* = number of postal areas

The Moran’s I statistic ranges from -1 to 1, with a positive value indicating that positive correlation (i.e. spatial clustering) is present. A negative value indicates that dispersion (or a competitive force) is present. Kelejian and Pruchal [19] demonstrate that Moran’s I statistic converges in distribution to a standard normal under the assumptions of a Tobit model. Therefore the Moran’s I can be used to test for spatial dependence in Tobit models. The degree of spatial correlation can be used to predict the degree to which variables and their spatial lags are correlated across the geographic plane of the data. For variables where spatial correlation is strong (i.e. Moran’s I larger than 0.7), the mean regionally weighted value (i.e. the mean of the value for the postal area and the mean of its neighbours) is used in an extended version of Eq. 1.

Region level variables are calculated using the following formula:

3 $$ {\tilde{x}}_{ri}=\frac{\left({x}_i+{\overline{x}}_i\right)}{2} $$

where: ${\tilde{x}}_{ri}$ = vector of variables measured at the region level

*x*_*i*_ = vector of variables in postal area *i*

${\overline{x}}_i\kern0.5em =\kern0.5em \frac{1}{n_i}\kern0.5em {\displaystyle \sum_j{w}_{ij}{x}_j}$, with *w*_*ij*_ = 0 if *i* = *j*

where: *n*_*i*_ = number of neighbours of *i*

The relationship between GP supply and area characteristics is estimated using two model specifications. The first is a Tobit model, which does not take account of the spatial correlation of the area (Eq. 1). The second is a spatially weighted Tobit model (Eq. 4), which takes account of both local area and neighbouring area values^1^:

4 $$ {S}_i={\alpha}_0+{x}_i\beta +{\overline{x}}_i\delta +{\tilde{x}}_{ri}\gamma +{u}_i,\kern0.5em \mathrm{where}\kern0.5em {u}_i\sim Normal\left(0,{\sigma}^2\right) $$

### Data

GP supply for 2008 is measured as the number of GPs per 1,000 persons. GP supply is calculated for each postal area using the main place of work for GPs in Australia. Data was purchased from Australasian Medical Directory maintained by the Australasian Medical Publishing Company [20]. Australian Bureau of Statistics (ABS) Census data for 2006 is use to capture the non-pecuniary area attributes [21]. Postal area is the lowest level of geographical aggregation in the data. Postal areas are approximately equivalent to a suburb or neighbourhood in urban areas and a region in rural areas. Postal areas in Australia have an average of approximately 8,700 residents but the numbers range widely from 56 - 85,333 residents [21].

Mean taxable income for each postal area was sourced from the Australian Tax Office [22] it is compiled using the aggregated individual income for postcodes. Labour force participation rates (percentage of the population who are employed or unemployed) were sourced from the ABS Census [21]. These are used to capture the socioeconomic status of an area. Socioeconomic status has long been found to be associated with health status [23]. For example, in the UK, economically deprived areas have been found to be relatively underserved [11].

Rural and remote locations are considered to be underserved in Australia. As a result, rurality has become a central focus of health care policies and political debate. Rurality is measured by the Accessibility/Remoteness Index of Australia (ARIA), which ranges from 0 being an urban centre to 15 being very inaccessible and remote [21].

Population health status is often used as a proxy for health care need. Areas with greater health care need are expected to have higher demand for services. The only direct measure of health status available in the Census is mortality and this is disaggregated to the Statistical Local Area (SLA) level only [21]. The spatial analysis will account for the fact that SLAs are slightly larger than postal areas. Health variables are often considered to be endogenous variables when measuring GP supply [12]. Nonetheless, mortality is used to capture population health and proxy health care need in this study. It is used as a time-lagged variable (i.e. GP supply is measured in 2008 and mortality rate in 2006) to reduce the likelihood of reverse causality.

Demographic factors such as age and gender are associated with consultation rates. Data from Australia show that females account for 57 % of all GP encounters, whereas females represent 50.4 % of the total population [24]. In 2013 patients aged over 65 years account for approximately 33 % of all encounters and represented only 14.4 % of the total population [24]. GP consultation rates show a U-shaped distribution by age, with children and the elderly seeking more frequent consultations than individuals in the middle of the age distribution [25, 26].

Another demographic variable included is the proportion of the population that is indigenous (i.e. from Aboriginal or Torres Strait Islander descent) [21]. In Australia, indigenous populations have lower health status and life expectancy than the non-indigenous population. Given the health discrepancy between the indigenous population and the general population, the government has created indigenous health incentives programs. These programs aim to attract GPs to locate in areas with a high proportion of indigenous population [27].

The attractiveness of an area, in terms of amenities, is also expected to influence GP supply. Areas with more amenities, such as private schools, are expected to have more GPs per capita. The presence of hospitals will be positively related to GP supply if GPs consider them to be amenities or complements, and negatively related to GP supply if hospitals are substitutes or a source of competition. Benham et al. [4] suggest that physicians have a preference to be near hospitals, perhaps due to referral networks. However, evidence of this is not given. Including a hospital variable provides an opportunity to investigate this relationship. Local amenities are measured as the number of hospitals and private schools in the postal area which are sourced from publically available directories [28, 29].

## Results

The number of postal areas included in the analysis is 2032. The mean number of contiguous neighbours (postal areas that share a geographic border) is five. Seventy five percent of all postal areas have between three and seven neighbours, and 96 % of postal areas have less than ten [30]. Two matrices are used to capture the characteristics of surrounding neighbourhoods; these are based on a definition of a neighbour being the five and ten closest postal areas respectively, as measured centroid to centroid. Table 1 presents the descriptive statistics of the data included in the analyses.

**Table 1:** Descriptive statistics of factors associated with GP supply across postal areas, 2006

Variable	Source	Year	Mean	SD	Range
GP supply					
GP count	AMPCo	2008	10.023	14.760	(0, 118)
GPs per 1,000	AMPCo	2008/06	0.909	1.154	(0, 22.727)
Annual gross taxable income ($10,000)	ATO	2006	4.826	1.403	(2.881, 19.363)
Labour force participation (%)	Census	2006	73.188	6.945	(42.610, 97.480)
Mortality Rate (per 1,000 persons)	Census	2006	6.205	1.554	(2.4, 36.7)
Rurality					
ARIA	Census	2006	2.157	2.958	(1, 15)
Amenities					
Hospitals	Directory	2009	0.399	0.734	(0, 11)
Private Schools	Directory	2010	1.022	1.702	(0, 14)
Indigenous (%)	Census	2006	2.770	7.116	(0, 88.366)
Female (%)	Census	2006	49.667	2.524	(24.55, 59.23)
Percent 0-14 (%)	Census	2006	19.909	4.424	(0, 38.498)
Percent 65+ (%)	Census	2006	13.929	5.518	(0, 42.947)
Population (1,000s)	Census	2006	8.697	10.447	(0.056, 85.333)

**Fig. 1:**
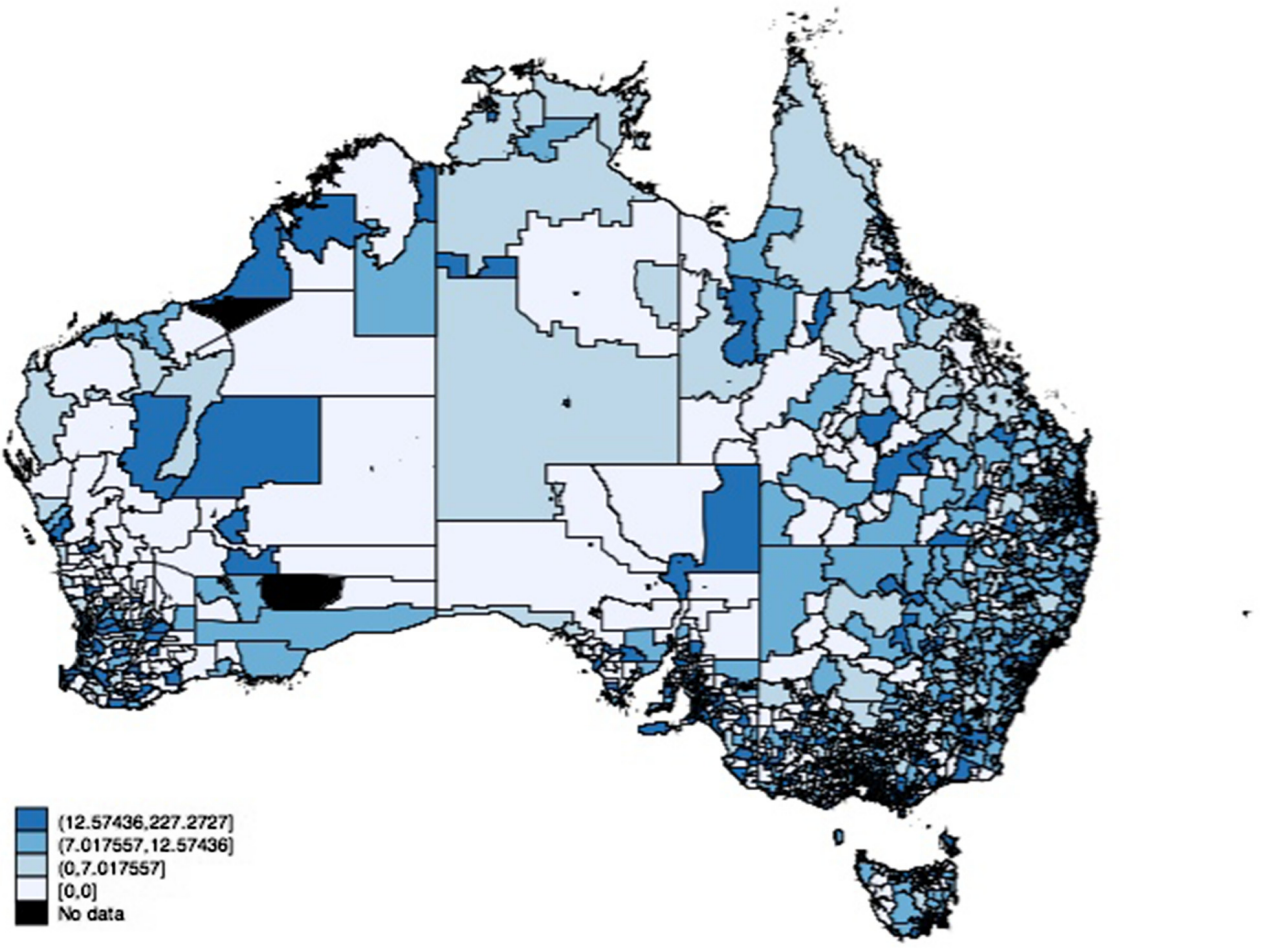
GP supply across Australia. Illustrates the supply of GPs per 1,000 persons across Australia. The figure demonstrates that there is considerable variation in the supply of GPs across postal areas in Australia. GPs supply is considerably higher in urban coastal areas compared to the more rural inland areas

**Fig. 2:**
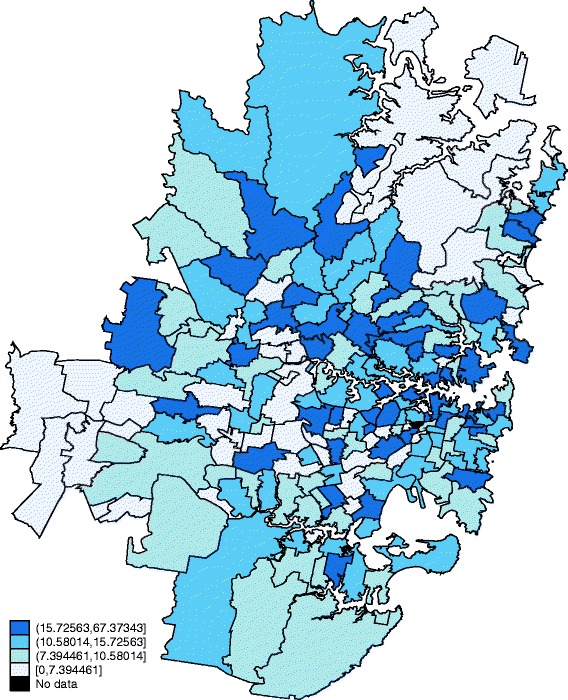
GP supply across Sydney. Illustrates the supply of GPs per 1,000 persons for the metropolitan area of Sydney. The figure demonstrates that some very populated metropolitan postal areas have low GP supply levels

**Fig. 3:**
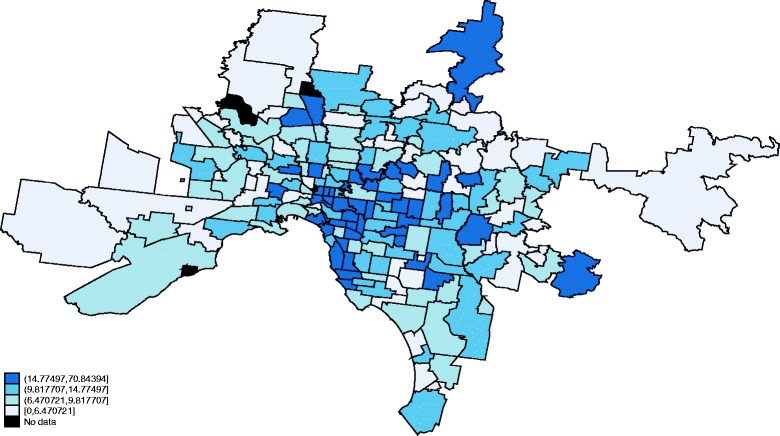
GP supply across Melbourne. Illustrates the supply of GPs per 1,000 persons for the metropolitan area of Melbourne. The figure demonstrates that despite a higher overall supply level in metropolitan areas there is heterogeneity in the supply of GPs across postal areas

The descriptive statistics show that postal areas in Australia have a mean of slightly less than one GP per 1,000 persons residing in the postal area. Figure 1 graphically depicts GP supply in Australia. There is considerable variation in the supply of GPs across postal areas in Australia; with GPs supply being considerably higher in urban coastal areas compared to the more rural inland areas. Figures 2 and 3 depict GP supply across Sydney and Melbourne respectively. All the Figures suggest that GP supply is not spatially clustered with some very populated areas having low GP supply levels.

To test for spatial correlation, a Moran’s I test-statistic is calculated for each of the included variables. The Moran’s I test-statistic for the dependent variable is reported in the first row of Table 2. Neighbouring GP supply levels do not act as a competitive force, nor does it appear that GPs are strongly spatially clustered, as the test statistic is positive but low (~0.11).

**Table 2:** Moran’s I test statistics

Variable	Moran’s I^a^	
	N = 5	N = 10
GPs (per 1000 persons)	0.108	0.112
	(8.492)	(12.437)
Mean Annual Taxable Income ($10,000)	0.730	0.670
	(56.199)	(72.923)
Labour Force Participation	0.450	0.394
	(34.530)	(42.780)
Mortality rate	0.413	0.347
	(32.502)	(38.608)
Rurality (ARIA)	0.866	0.810
	(66.456)	(87.880)
Hospital	0.057	0.051
	(4.421)	(5.599)
Private Schools	0.218	0.206
	(16.757)	(22.385)
Indigenous (%)	0.457	0.389
	(35.561)	(42.764)
Females (%)	0.305	0.269
	(23.474)	(29.318)
Proportion under 14 (%)	0.495	0.445
	(37.957)	(48.302)
Proportion over 65 (%)	0.447	0.388
	(32.249)	(42.040)

Notes: ^a^all Moran’s I statistics are statistically significant

z-scores are presented in parentheses

Moran’s I statistics for the independent variables are reported in the remaining rows of Table 2. The results demonstrate that many of the independent variables are spatially correlated. Mean income and rurality have particularly high Moran’s I statistics indicating that the value for a given postal area is highly correlated to the mean value of their five or ten closest neighbours. In order to minimize multicollinearity in the spatial Tobit model the rurality and mean income are included at the regional level (computed using Eq. 3) with the remainder of the independent variables included both at the postal area and neighbourhood level (i.e. spatially lagged).

The results of the non-spatial Tobit model are presented in column two of Table 3. Tobit models accounting for spatial heterogeneity by including lags of the neighbours’ values are presented in columns three (weighted with the five closest neighbours) and four (weighted with the ten closest neighbours) of Table 3.

**Table 3:** Non-spatial and spatial Tobit models

	Tobit	Spatial Tobit	Spatial Tobit
		*N* = 5	*N* = 10
Mean Income ($10,000)^d^	0.078^c^	0.084^b^	0.069^a^
	(0.026)	(0.035)	(0.038)
Labour Force Participation Rate (%)	0.027^c^	0.034^c^	0.034^c^
	(0.008)	(0.009)	(0.009)
Neighbouring Labour Force Participation Rate (%)		−0.037^c^	−0.037^b^
		(0.014)	(0.017)
Mortality Rate (%)	0.034	0.014	0.010
	(0.026)	(0.028)	(0.028)
Neighbouring Mortality Rate (%)		0.015	0.011
		(0.053)	(0.068)
ARIA score^d^	−0.085^c^	−0.061^b^	−0.067^b^
	(0.017)	(0.025)	(0.029)
Hospitals (count)	0.380^c^	0.377^c^	0.385^c^
	(0.046)	(0.046)	(0.046)
Neighbouring Hospitals (count)		−0.015	−0.116
		(0.106)	(0.144)
Private Schools (count)	0.094^c^	0.075^c^	0.072^c^
	(0.020)	(0.021)	(0.021)
Neighbouring Private Schools (count)		0.091^b^	0.146^c^
		(0.039)	(0.047)
Indigenous (%)	0.023^c^	0.012^a^	0.011
	(0.007)	(0.007)	(0.007)
Neighbouring Indigenous (%)		0.012	0.026
		(0.013)	(0.016)
Female (% of population)	0.075^c^	0.057^c^	0.063^c^
	(0.017)	(0.019)	(0.018)
Neighbouring Female (% of population)		0.047	0.020
		(0.032)	(0.039)
Proportion over 65 (% of population)	0.019^a^	0.054^c^	0.051^c^
	(0.011)	(0.013)	(0.013)
Neighbouring Proportion over 65 (% of population)		−0.086^c^	−0.082^c^
		(0.020)	(0.023)
Proportion under 14 (% of population)	−0.093^c^	−0.071^c^	−0.076^c^
	(0.009)	(0.013)	(0.013)
Neighbouring Proportion under 14 (% of population)		−0.031^a^	−0.031^a^
		(0.017)	(0.018)
Constant	−4.230^c^	−2.271	−1.572
	(1.039)	(1.880)	(2.387)
Log likelihood	−2872.085	−2852.798	−2852.942
Link-test hat-squared	−0.221^c^	−0.250^c^	−0.249^c^
	(0.033)	(0.032)	(0.032)
Moran’s I		0.029^c^	0.049^c^
z-score		2.349	5.450
Observations	2032	2032	2032

Variable coefficients with standard errors in parentheses

^a^significant at the 10% level; ^b^significant at the 5% level; ^c^significant at the 1% level

^d^measured at the regional level for the spatial models (Eq. 3)

The results of the non-spatial Tobit model demonstrate that postal areas with higher incomes are associated with higher GP supply. Higher labour force participation rates are also positively associated with GP supply. The rurality measure, ARIA, has a statistically significant and negative association with GP supply. Amenities such as the number of hospitals and private schools have statistically significant positive associations with GP supply. With regards to demographic factors, females and the proportion of the population over 65 are associated with higher GP supply and the proportion of the population under 14 is negatively associated with GP supply. Postal areas with higher indigenous populations have a positive association with GP supply. Mortality is not related to GP supply.

The spatially weighted Tobit models provide a slight improvement in explanatory power compared to the original Tobit. The log-likelihood values are closer to zero for the spatial Tobit models. However, due to income and rurality being measured at the regional level, these models are not nested; and comparisons should therefore be interpreted with caution.

## Discussion

This paper has investigated the effect that nearby postal areas have on GP supply in a given postal area. Including a neighbourhood value (a spatially weighted parameter for the mean value of the five and ten closest neighbours) adds to the fit of the model for several of the variables. Therefore the hypothesis that geographic and demographic factors of nearby neighbourhoods are associated with the GP supply in a particular neighbourhood cannot be rejected.

However, neighbouring factor effects are not uniform across the included variables. In some instances neighbouring factors act as a negative spill-over with strong postal-area-specific forces for attracting GP supply. Postal areas whose neighbours have high labour force participation rates and more elderly persons are associated with lower GP supply, indicating that GPs are more likely to locate directly in (rather than in close proximity to) neighbourhoods with these factors. This result is consistent with literature suggesting that patients travel very small distances to consult a GP. In Australia, a study by Hyndman et al. [31] demonstrated that patients travelled a median distance of 4.3km in poor access areas and 3.1km in high access areas to see their chosen GP. Demographic characteristics are therefore considered to be postal-area-specific factors. However, postal areas and neighbouring postal areas with higher proportions of persons under 14 are negatively associated with GP supply. Perhaps these are newer suburbs, where young families tend to locate, without established GP clinics. Conversely, neighbouring factors can also act as positive spill-overs. For instance, GP supply is not only higher in postal areas where there are more private schools, but also in postal areas where the neighbours have more private schools. In fact the neighbouring effect of private schools is stronger than the postal-area-specific effect, possibly because GPs reside in areas close to where they work, therefore GPs are locating their practice near private schools, but not necessarily in the same postal area.

The number of hospitals is significantly and positively related to GP supply in all model specifications. Hospitals appear to be a leading factor with regard to high GP supply. As hypothesized by Benham et al. [4], GPs appear to consider hospitals to be complementary amenities. It is also possible that postal areas with hospitals may have higher demand for GPs due to other unmeasured location factors, such as proximity to the city centre or transport, which may also be attractive to GPs, resulting in GPs locating in the same postal area as hospitals.

As expected, GP supply is negatively associated with regional rurality score (ARIA) and positively related to area mean income. Areas with higher mean incomes may provide better working conditions and are therefore attractive to GPs. One limitation of this study is that data on the price of GP services was not available, therefore it is plausible that the relationship between mean income of an area and GP supply is indicating that GPs are charging higher fees in these areas. However, the relationship between location choice, GP supply and fees is complex [32, 33]. Income is highly spatially correlated. Since the analysis is based on location of work, it is possible that this result is attributable to GPs working in one postal area but residing in a nearby postal area.

The variable capturing the proportion of indigenous persons is significant in the non-spatial Tobit model and the 5-neighbour band, but not significant in the 10-neighbour band. This could signal that this variable is associated with an omitted variable that is spatially related. Therefore, conclusions regarding the relationship between GP supply and the proportion of indigenous persons in the postal area are limited. Nonetheless, this finding demonstrates that without accounting for spatial correlation erroneous conclusions regarding the effectiveness of policies could be drawn.

According to Smith et al. [34], areas with a high under-65 mortality rate are among the areas with the highest health care need. Mortality rate is not significantly associated with GP supply. If the assumption that mortality rate is exogenous to GP supply is correct, then this suggests that GPs are not located in accordance with health care needs (over and above responding to the age and gender mix of the population). However, understanding this relationship would require the use of other population health measures, specifically those capturing morbidity, which were not available.

The results demonstrate that the level of geographic aggregation is an important consideration when addressing the distribution of GPs. The strength of the association between geographically measured factors and GP supply depends on the level at which the variables are measured. Allowing for flexibility in the geographic level at which explanatory factors are aggregated has been presented as a potential solution to the limitations imposed by arbitrary geographic boundaries imposed by geographically referenced GP supply data.

Although the spatial approach presented in this paper is an improvement on ignoring the spatial dimensions of the data, it is not without its limitations. One of the limitations of adopting a spatial approach is that in order to add in the spatial dimension a symmetric spatial weights matrix needs to be defined. This limits the analysis to the selection of a pre-specified number of neighbours rather than a pre-specified distance or a more flexible approach of the actual number of contiguous neighbours. This is clearly another (albeit purposely exogenous) arbitrary delineation of space. It is nevertheless a step toward understanding the role that the geographic nature of GP supply data play in analyses of this nature.

Although GPs may be located where there is demand (i.e. a high proportion of elderly and females of child-bearing age) they are not necessarily locating in areas that are considered to have the highest health care needs (i.e. areas with high mortality rates, low socioeconomic status, or in rural areas). If horizontal equity is a health care priority, then policies aimed to redistribute GPs into these areas are essential.

## Conclusion

Although GP supply is not spatially correlated, many locational factors that are associated with GPs’ supply are spatially correlated. Therefore the level on which geographic variables are being considered by GPs is unlikely to match the arbitrary boundaries imposed by the data. Many variables, such as those related to demand, are neighbourhood specific. However, GP supply also appears to be associated with factors beyond the boundaries of the direct neighbourhood where they practice. These would be missed if spill-over effects were not considered.

## Footnotes

1 Alternative model specifications (i.e. negative binomial and OLS) have also been estimated and provide similar results to the Tobit model presented in this paper.
